# Gender Disparities in Healthcare Utilization and Expenditures Among Single-Parent Households in Korea

**DOI:** 10.3390/healthcare14080976

**Published:** 2026-04-08

**Authors:** ShinYoung Kim, Jinhyung Lee

**Affiliations:** 1 Division of Macroeconomic and Financial Policy, Korea Development Institute (KDI), Sejong 30149, Republic of Korea; michelle7111@naver.com; 2 Department of Economics, Sungkyunkwan University, Seoul 03063, Republic of Korea

**Keywords:** health inequality, universal health coverage, family structure, single-parent households, gender disparities, healthcare utilization, out-of-pocket expenditure

## Abstract

**Highlights:**

**What are the main findings?**

- Healthcare disparities differ across stages of care: single fathers face disadvantages at the initiation stage, whereas single mothers experience greater disadvantages primarily after access to care.
- Similar probabilities of healthcare utilization do not ensure equal financial protection; single mothers bear the greatest expected medical expenditure due to the combination of higher unmet healthcare needs and higher conditional spending.

**What are the implications of the main findings?**

- Similar probabilities of healthcare use do not ensure equitable financial protection; disparities are observed across access and post-access stages.
- Universal coverage must move beyond formal entitlement and incorporate targeted, gender-sensitive policies that mitigate structural vulnerabilities within single-parent households.

**Abstract:**

**Background/Objectives**: This study examines gender differences in healthcare utilization and financial burden across family structures under Korea’s near-universal health insurance system. **Methods**: Using 2010–2018 Korea Health Panel data, we applied a two-part model to estimate initiation of care, conditional utilization, and expected out-of-pocket expenditures. **Results**: Single fathers were less likely to initiate care, whereas single mothers had higher unmet needs and substantially greater conditional and expected out-of-pocket spending, with expected expenditures approximately 46% higher than those of two-parent households. **Conclusions**: We document stage-specific disparities in healthcare utilization and financial burden across family structures even under near-universal coverage, indicating the need for policies that strengthen both access and financial protection for single-parent households.

## 1. Introduction

Reducing health inequality has become a central concern in global health policy and social science research. Although many countries have expanded universal health coverage (UHC) to improve access to care, substantial disparities in healthcare utilization, unmet medical needs, and financial burden persist. Comparative research shows that formal insurance coverage alone does not eliminate inequality; rather, structural determinants—such as socioeconomic position, labor market precarity, and household organization—continue to be associated with who accesses care and under what conditions [1].

Within this broader debate, family structure has been recognized as an important dimension of health stratification. Across high-income countries, single-parent households face elevated risks of poverty, unstable employment, and psychosocial stress. These constraints may translate into delayed care, unmet medical needs, and disproportionate out-of-pocket burdens. Evidence from Europe and North America suggests that lone-parent families experience systematically worse health outcomes and greater barriers to healthcare than two-parent households [2,3]. These findings suggest that healthcare inequality operates not only through income gradients but also through the interaction of caregiving burden, time scarcity, and gendered labor market positions.

Despite growing international attention, little is known about how healthcare utilization differs by family structure within East Asian universal insurance systems. Korea presents a particularly informative case. Having achieved near-universal health insurance coverage, Korea is often cited as a model of rapid UHC expansion. Nevertheless, disparities in healthcare utilization and unmet needs remain across socioeconomic groups. At the same time, single-parent households account for more than 10% of all families and continue to increase in prevalence [4]. Mother-headed households, in particular, experience lower income levels, unstable employment, and concentrated caregiving responsibilities [5]. These structural vulnerabilities raise a critical question: whether healthcare utilization and financial burden differ systematically across gendered family types even under universal coverage.

This study has three main aims. First, it conceptualizes family structure as an important dimension of healthcare inequality and distinguishes between single mothers and single fathers to identify gender-differentiated patterns. Second, it employs a two-part modeling framework to separate the probability of healthcare utilization from conditional medical expenditures, thereby examining whether observed disparities are more pronounced at the access stage or at the spending stage. Third, by estimating expected medical expenditures, the study provides a comprehensive measure of financial burden and offers new evidence on the limits of universal health coverage in addressing gendered and family-based inequality.

Using nationally representative Korea Health Panel data from 2010 to 2018, this study examines healthcare utilization, unmet healthcare needs, utilization intensity, and out-of-pocket expenditures among single-parent and two-parent households. By situating the Korean case within the broader international debate on social determinants and universal coverage, the findings deepen understanding of how healthcare inequality differs across gendered family structures. Health inequality research increasingly emphasizes structural determinants rather than individual-level characteristics. The behavioral model of healthcare utilization conceptualizes service use as the interaction of predisposing characteristics, enabling resources, and need factors [6]. Subsequent refinements highlight that enabling resources—such as income stability, employment security, and household support—are unequally distributed across social groups, producing systematic disparities in access to care [7]. From this perspective, family structure functions as a structural context that conditions both economic resources and caregiving obligations.

The social determinants framework further underscores that inequalities persist even in universal healthcare systems because socioeconomic stratification shapes exposure to stress, resource scarcity, and time constraints [1]. Single-parent households are particularly exposed to these pressures. Economic precarity combined with concentrated caregiving responsibility may create both financial and non-financial barriers to healthcare access. Non-monetary constraints—such as time scarcity and work–family conflict—can limit the ability to initiate care, while limited financial reserves increase vulnerability to out-of-pocket burdens once care is accessed.

Comparative research supports this multi-mechanism perspective. Lone-parent households are disproportionately represented among the poor, and poverty is strongly associated with adverse health outcomes [2]. In market-oriented healthcare systems, single mothers face higher risks of cost-related delays and catastrophic medical expenditure [3,8]. Importantly, these patterns persist even after insurance expansion, indicating that universal entitlement does not automatically ensure equitable financial protection.

Gender may condition how these structural constraints are associated with healthcare experiences. Cross-national studies show that women disproportionately shoulder unpaid caregiving responsibilities, increasing psychosocial stress and opportunity costs associated with seeking care [9]. Single fathers, meanwhile, may experience weaker informal support networks and distinct institutional navigation barriers [10]. These differentiated constraints suggest that healthcare utilization patterns within single-parent households are unlikely to be homogeneous and must be analyzed through a gender-sensitive lens.

In Korea, scholarship has documented the socioeconomic precarity of single-parent families. Pronounced income disparities [11,12], concentration in precarious employment, and elevated poverty risks characterize this population [13,14,15]. Subjective health disadvantages among single mothers are closely linked to economic insecurity and caregiving stress [16]. However, while these studies identify structural vulnerability, they do not systematically connect these conditions to stage-specific healthcare utilization outcomes.

Research on healthcare inequality in Korea has primarily examined income gradients and employment status. Employment instability reduces healthcare utilization and medical spending, and income-related inequities persist when utilization is assessed conditional on need [17,18]. Yet family structure remains largely absent from these analyses. Moreover, most empirical studies rely on single-equation models that implicitly assume a unified decision process. Health econometric research demonstrates that healthcare utilization involves distinct stages—initiation and conditional spending—and that modeling these stages separately is essential to avoid biased inference [19,20].

Despite extensive international evidence on structural determinants of health and substantial documentation of socioeconomic vulnerability among Korean single-parent families, no prior study has simultaneously examined (1) gender-differentiated patterns within single-parent households, (2) stage-specific disparities in healthcare utilization, and (3) expected medical expenditure as a comprehensive measure of financial burden under universal insurance. Addressing these omissions requires integrating family structure and gender into a multi-stage analytical framework of healthcare utilization.

## 2. Materials and Methods

### 2.1. Data

The data used in this study are drawn from the Korea Health Panel (KHP), a nationally representative longitudinal survey jointly administered by the Korea Institute for Health and Social Affairs and the National Health Insurance Service. The KHP provides comprehensive information on healthcare utilization, medical expenditures, insurance coverage, and socioeconomic characteristics of Korean households. This study utilizes nine annual survey waves spanning 2010 to 2018. The panel design enables the analysis of repeated observations over time and enhances the reliability of healthcare utilization measures.

The KHP collects detailed information on whether individuals used healthcare services during the reference period, including the frequency of outpatient visits, inpatient admissions, and experiences of unmet healthcare needs. In addition, the survey provides rich demographic and socioeconomic information, such as household composition, income, employment status, educational attainment, insurance coverage, and self-rated health status. These variables allow for a multidimensional assessment of healthcare access and financial burden within diverse family structures.

The KHP contains both household-level and person-level information. In this study, we use the person-level panel file and construct all outcome variables from individual records rather than household aggregates. Accordingly, healthcare utilization and medical expenditure measures refer to the sampled parent’s own person-level record, not to household-level or child-level utilization.

### 2.2. Study Population

The analytic sample was restricted to adults aged 19 years and older who were identified as parents residing with their children. The sample includes married fathers and mothers in two-parent households as well as single fathers and single mothers heading single-parent households; in two-parent households, both parents are included in the analytic sample when identified as the household head or spouse in the person-level file. Because the unit of analysis is the individual-year observation, all utilization and expenditure outcomes are interpreted at the parent level. That is, the study examines parental healthcare use among adults living with children, rather than children’s healthcare utilization. No upper age limit was imposed in order to capture the full spectrum of healthcare needs among adult parents.

To ensure data quality and consistency across model specifications, observations with missing or invalid responses on key variables—including healthcare utilization, educational attainment, self-rated health status, and household income—were excluded from the analysis. After applying these restrictions, the final analytic sample comprised 53,309 individuals from two-parent households and 4577 individuals from single-parent households across the pooled panel waves. The unit of analysis is the individual-year observation.

### 2.3. Measurement of Variables

The variables examined in this study are summarized in Table 1. Consistent with the study’s multi-stage analytical framework, the dependent variables capture distinct dimensions of healthcare access, utilization intensity, and financial burden.

#### 2.3.1. Dependent Variables

First, healthcare utilization was defined as a binary indicator equal to 1 if the sampled parent had positive individual-level medical expenditure during the reference period, and 0 otherwise. This measure captures whether any parent-level healthcare use associated with positive individual medical spending occurred during the observation period. Because this variable is constructed from the person-level medical expenditure record, it does not measure children’s healthcare utilization or household-level utilization as a whole. Given that Korea has one of the highest outpatient visit rates among OECD countries—averaging 15.7 visits per capita annually—the decision to initiate care represents a meaningful behavioral margin rather than a rare event [21].

Second, unmet healthcare needs were measured as a binary variable indicating whether respondents reported needing medical examination or treatment during the past 12 months but being unable to obtain it. Following Carr and Wolfe, individuals answering “yes” to this survey question were classified as having unmet healthcare needs [22]. This measure captures qualitative barriers to effective access that may persist despite formal insurance coverage.

Third, outpatient visits were measured using the number of outpatient visits recorded for the sampled parent in the person-level file. Inpatient admissions were measured using the corresponding person-level inpatient-use measure. These variables are defined at the individual parent level and are interpreted as parent-level utilization measures in the analysis.

Fourth, healthcare expenditures were defined as annual out-of-pocket medical spending incurred by individuals. Because expenditure data are right-skewed and strictly positive among users, medical expenditures were modeled using a gamma GLM with a log link.

#### 2.3.2. Independent Variable

The primary independent variable is family structure. Based on household composition and parental status, respondents were categorized into three mutually exclusive groups: (1) two-parent households, (2) single-father households, and (3) single-mother households. Two-parent households are more likely to share caregiving and income responsibilities, whereas single-parent households concentrate both roles in a single individual. These structural differences are likely associated with disparities in economic resources, time availability, and caregiving burden, which may in turn be related to patterns of healthcare utilization and financial exposure.

#### 2.3.3. Control Variables

Control variables were selected based on the behavioral model of healthcare utilization, which distinguishes among predisposing, enabling, and need factors [6]. Organizing covariates within this framework enables systematic assessment of whether disparities by family structure persist after accounting for socioeconomic resources and health needs.

##### Predisposing Factors

Predisposing factors refer to demographic and sociocultural characteristics that exist prior to the onset of illness and influence health behavior. In this study, age and educational attainment were included. Age was calculated from respondents’ year of birth and treated as a continuous variable. Educational attainment was categorized into four groups: elementary school or below, middle school graduate, high school graduate, and college or above. Education serves as a proxy for socioeconomic status and health literacy, which may shape healthcare-seeking behavior [23].

##### Enabling Factors

Enabling factors capture the economic and structural resources that facilitate or constrain access to care. These include household size, annual household income, and private health insurance coverage. Household size reflects the distribution of economic and caregiving resources within the household and may affect time availability for seeking care. Annual household income, defined as total labor and asset income earned during the previous year, was included as an indicator of financial capacity. Private health insurance coverage was measured as a binary variable indicating whether the respondent held supplementary private insurance, which may reduce out-of-pocket risk.

##### Need Factors

Need factors represent the most immediate determinants of healthcare utilization, reflecting individuals’ underlying health demand. Self-rated health status was measured on a five-point scale ranging from very good to very poor. Self-rated health is widely recognized as a strong predictor of mortality and healthcare utilization [24]. In addition, limitations in daily activities were measured based on whether respondents reported difficulties in performing routine activities such as work, study, or household tasks. This variable captures functional impairment and serves as a direct indicator of medical need.

### 2.4. Statistical Analysis

This study aims to examine whether healthcare disparities associated with family structure are more evident at the stage of initiating care, at the stage of utilization intensity and spending, or at both stages. To capture these potentially distinct patterns, we employ a two-part modeling framework that separates the decision to use healthcare from the level of utilization and expenditures conditional on use.

Healthcare utilization data are characterized by a large proportion of zero observations and a right-skewed distribution of expenditures. A single-equation regression approach would fail to adequately account for this structure. Following Manning et al., we adopt a two-part model in which the first stage estimates the probability of any healthcare use, and the second stage examines utilization intensity and medical expenditures among users [19].

In the first stage, we estimate a logistic regression model in which the dependent variable is a binary indicator of whether an individual used any inpatient or outpatient services during the previous year. The key explanatory variable is family structure (two-parent households, single fathers, and single mothers), and all models control for predisposing, enabling, and need factors based on the Andersen framework [6].

In the second stage, conditional on positive utilization, we model the frequency of outpatient visits and inpatient admissions using negative binomial regression to account for overdispersion in count data. Medical expenditures are estimated using a generalized linear model (GLM) with a log link and Gamma distribution, consistent with the skewed and strictly positive nature of healthcare spending.

To provide a comprehensive measure of financial burden, we compute individual-level expected out-of-pocket medical expenditures as:

*E*(*y*∣*x*) = Pr(*y* > 0∣*x*) × *E*(*y*∣*y* > 0,*x*)

where E(y|x) denotes expected out-of-pocket medical expenditure, Pr(y>0|x) denotes the predicted probability of positive medical expenditure, E(y|y>0,x) denotes the predicted conditional mean expenditure among those with positive expenditure, y represents out-of-pocket medical expenditure, and x denotes the vector of covariates included in the model. That is, expected expenditure is calculated as the product of the predicted probability of any positive expenditure and the predicted conditional mean expenditure among those with positive expenditure. This decomposition allows us to determine whether observed disparities in expected spending reflect differences in the probability of positive expenditure, spending intensity conditional on positive expenditure, or both.

Because the data are longitudinal, we assessed the suitability of fixed- and random-effects specifications using Hausman tests comparing conditional fixed-effects logit and random-effects logit models. The tests indicated statistically significant differences for healthcare utilization and unmet healthcare needs, suggesting potential correlation between unobserved individual heterogeneity and the covariates. However, the key independent variable—family structure—exhibits minimal within-individual variation over time. Consequently, fixed-effects estimation substantially reduces the effective sample and primarily captures within-individual transitions, limiting its usefulness for estimating between-group differences across family types. Given that the study’s objective is to examine disparities across family structures rather than within-individual changes, random-effects models are retained as the main specification. Fixed-effects estimates are reported in Appendix A Table A1 as a robustness check. All statistical analyses were conducted using Stata 19 (StataCorp LLC, College Station, TX, USA).

## 3. Results

### 3.1. Descriptive Statistics

Table 1 summarizes demographic characteristics and healthcare utilization patterns across two-parent and single-parent households by gender. Differences in both utilization and socioeconomic conditions are evident.

Regarding healthcare utilization, 13.79% of individuals in two-parent households reported no medical use during the reference year. The non-utilization rate was slightly higher among single fathers (15.43%) but lower among single mothers (11.98%), indicating comparatively higher utilization among the latter. Despite these patterns, unmet healthcare needs were substantially more prevalent in single-parent households. Specifically, 21.90% of single mothers and 18.96% of single fathers reported unmet needs, compared with 13.64% in two-parent households. Thus, higher or comparable utilization does not correspond to lower unmet need among single-parent families.

Utilization intensity further underscores these disparities. Average outpatient visits were considerably higher among single mothers (24.87) and single fathers (27.24) than among two-parent households (14.69). Inpatient admissions followed a similar pattern, with single mothers reporting the highest average (1.85), followed by single fathers (1.43) and two-parent households (1.38). Out-of-pocket expenditures mirrored these trends: single mothers incurred the highest average spending (approximately 546 USD), followed by single fathers (approximately 448 USD) and two-parent households (approximately 375 USD).

Marked socioeconomic differences accompany these utilization patterns. Single-parent respondents were roughly ten years older on average than those in two-parent households. Educational attainment was substantially lower among single parents, particularly single mothers, among whom 34.51% had elementary education or below. Income disparities were pronounced, with average annual household income nearly twice as high in two-parent households (approximately 38,317 USD) as in single-mother households (approximately 20,350 USD). Household size also differed, with single-parent families predominantly consisting of two to three members.

Insurance coverage and health status indicators further reflect structural vulnerability. Private insurance enrollment was highest in two-parent households (85.12%) and lowest among single fathers (58.51%). Single mothers reported poorer self-rated health and higher rates of activity limitations than their counterparts in two-parent households.

Overall, the descriptive evidence suggests that single-parent households—particularly those headed by women—are older, economically disadvantaged, and in poorer health, while simultaneously exhibiting higher utilization intensity and greater unmet healthcare needs. These patterns provide preliminary evidence of structural health inequality that is further examined in the multivariate analysis.

### 3.2. Effects on Healthcare Utilization and Unmet Healthcare Needs

Table 2 presents the first-stage logit estimates of healthcare utilization and unmet healthcare needs by family structure. Compared with individuals in two-parent households, single fathers were significantly less likely to utilize healthcare services (OR = 0.50, *p* < 0.05), suggesting barriers at the initiation stage. However, no statistically significant difference was observed in unmet healthcare needs for single fathers. These first-stage results suggest that single fathers may face greater difficulty at the point of initiating care.

In contrast, single mothers did not differ significantly from two-parent households in the probability of healthcare utilization, but were significantly more likely to report unmet healthcare needs (OR = 1.38, *p* < 0.01). This pattern suggests that while formal entry into the healthcare system may be comparable for single mothers, effective access remains constrained.

Control variables followed theoretically consistent patterns. Age was positively associated with healthcare utilization and negatively associated with unmet healthcare needs. Higher educational attainment was linked to lower utilization and reduced unmet needs. Larger household size was associated with lower utilization but higher unmet needs, suggesting potential resource constraints within larger households.

Household income exhibited a conventional gradient: higher income increased the likelihood of healthcare utilization and decreased the probability of unmet healthcare needs. Private health insurance coverage was positively associated with utilization but showed no significant relationship with unmet healthcare needs.

Health status indicators displayed strong and monotonic associations with both outcomes. As self-rated health deteriorated, the likelihood of healthcare utilization and unmet healthcare needs increased substantially. Individuals reporting very poor health were more than five times as likely to utilize services and more than six times as likely to experience unmet needs compared with those reporting very good health. This pattern reflects the strong role of medical need in shaping both care-seeking behavior and residual unmet demand.

Overall, the findings reveal differentiated gender patterns within single-parent households. Single fathers face disadvantages primarily at the initiation stage of care, whereas single mothers experience elevated unmet healthcare needs despite similar utilization probabilities. These stage-specific disparities motivate further examination of whether differences are also observed at subsequent stages of healthcare utilization intensity and medical expenditures.

### 3.3. Healthcare Utilization Frequency and Expenditures

Table 3 reports the second-stage estimates of the two-part model, examining utilization intensity, medical expenditures, and unmet healthcare needs among individuals with positive healthcare use.

Conditional on accessing care, distinct patterns emerged across family types. Single fathers had significantly more outpatient visits (β = 0.20), and single mothers also showed a smaller but statistically significant increase (β = 0.05), indicating higher outpatient utilization among users in single-parent households. Combined with the first-stage estimates, this suggests that single fathers were less likely to initiate healthcare use but had more outpatient visits once care was accessed. For inpatient admissions, only single mothers exhibited significantly higher hospitalization levels (β = 0.18), while no difference was observed for single fathers.

Regarding medical expenditures, single mothers incurred higher out-of-pocket spending (β = 0.09, *p* < 0.05), whereas single fathers did not differ significantly from individuals in two-parent households. Thus, among healthcare users, elevated financial burden appears concentrated primarily among single mothers.

Control variables displayed consistent and theoretically coherent associations. Older age and poorer self-rated health were strongly associated with higher outpatient visits, inpatient admissions, and medical expenditures. Higher educational attainment was generally linked to fewer outpatient visits and lower spending. Larger household size was not uniformly associated with inpatient admissions; only households with five or more members exhibited significantly higher inpatient use. Functional limitations were positively associated with both utilization intensity and spending.

Unmet healthcare needs were also examined among users. Even after initiating care, single mothers remained significantly more likely to report unmet needs, indicating that disparities persist beyond the initial access stage.

Taken together, the second-stage findings reinforce the stage-specific gender divergence identified in the first-stage analysis. While single fathers primarily face barriers at the point of initiation, single mothers experience greater utilization intensity, higher financial burden, and continued unmet needs once care is accessed. These results suggest that disparities among single-parent households may differ across stages of healthcare utilization.

### 3.4. Expected Medical Expenditures by Family

Table 4 presents the decomposition of expected out-of-pocket medical expenditures by family structure. Expected expenditure is calculated as:

(1) E(Y)=P(Use=1)×E(Y∣Use=1)

where P(Use=1) denotes the predicted probability of any healthcare utilization and E(Y∣Use=1) represents the predicted conditional mean expenditure among users. This formulation allows overall financial burden to be decomposed into an access component and a conditional spending component.

To construct Table 4, we first estimated the probability of positive medical expenditure for each individual using the first-part random-effects logit model. We then estimated conditional mean expenditure among individuals with positive expenditure using the second-part gamma generalized linear model with a log link, and generated predicted values for the full analytic sample. Individual-level expected expenditure was calculated as the product of these two predicted components, and the values reported in Table 4 are group averages of the resulting individual-level predicted values.

Although predicted utilization probabilities were broadly similar across groups (86.66% for two-parent households, 86.33% for single fathers, and 90.58% for single mothers), conditional mean expenditures differed substantially. Single mothers incurred the highest predicted spending among users (USD 607.85), followed by single fathers (USD 574.13), and two-parent households (USD 433.79).

When combining these components, expected medical expenditures were USD 561.90 for single mothers, USD 507.91 for single fathers, and USD 384.22 for two-parent households. Relative to two-parent households, expected expenditures were approximately 46% higher for single mothers and 32% higher for single fathers. These differences appear to reflect variation in conditional spending rather than utilization probability.

From a financial protection perspective, these findings suggest that universal coverage does not equalize effective economic risk exposure. Despite comparable probabilities of healthcare use, single-parent households—particularly single mothers—remain disproportionately exposed to post-access financial burden. The elevated expected expenditure among single mothers is consistent with a cumulative disadvantage: high utilization combined with higher conditional spending.

This pattern is consistent with a structural limitation of universal health insurance systems operating with substantial cost-sharing requirements. Formal coverage may secure entry into the healthcare system, but it does not necessarily shield vulnerable households from intensified financial strain once care is accessed.

Taken together, the results in Section 3 indicate that disparities across family types are observed not only in healthcare utilization but also in out-of-pocket spending, with single-mother households facing the greatest overall financial burden.

## 4. Discussion

### 4.1. Policy Implications

This study shows that healthcare utilization and financial burden differ by family structure even within a universal health insurance system. By applying a two-part modeling framework, we distinguish disparities arising at the initiation of care from those emerging at the conditional utilization and spending stage, thereby highlighting stage-specific patterns that conventional single-equation models may obscure.

The Korean healthcare system illustrates a form of universalism without complete financial protection. Although National Health Insurance coverage is nearly universal, substantial cost-sharing requirements and reliance on supplementary private insurance generate unequal patterns of effective access. Under such institutional arrangements, formal coverage does not eliminate differences in financial risk exposure. Single-parent households have lower private insurance enrollment than two-parent households; among single parents, enrollment is particularly low for single fathers, while single mothers also remain below two-parent households. In this institutional context, cost-sharing may be especially consequential for single-parent households because medical spending competes more directly with other essential household expenditures. The limited role of the NHI benefit package in fully shielding households from out-of-pocket costs means that supplementary private insurance can function as an important buffer against post-access financial burden. When private insurance coverage is less prevalent, as in single-parent households, formal entitlement to care may not translate into equal financial protection.

Our findings extend the social determinants literature by showing that disparities associated with family organization persist under universal coverage and appear to differ across stages of healthcare use. Rather than reflecting a single unified decision process, healthcare disparities appear across multiple stages of care.

Gender-differentiated patterns are especially pronounced. Single fathers are significantly less likely to initiate healthcare utilization, indicating barriers at the access stage. Prior studies generally report that women use more outpatient and other non-acute healthcare services than men, while some prior evidence has also reported more frequent physician contact among single fathers than among couple fathers [25,26]. Taken together, our findings point to a stage-specific pattern rather than a simple reversal of the usual gender pattern: single fathers appear disadvantaged in initiating care yet show higher outpatient use once care is accessed. These barriers may reflect time constraints, weaker informal support networks, or institutional navigation challenges. In contrast, single mothers exhibit comparable probabilities of initiating care but face substantially higher unmet healthcare needs and greater conditional expenditures once care is accessed. This pattern is consistent with prior research showing greater unmet need and vulnerability among single mothers relative to partnered mothers [25,26]. However, these patterns should not be interpreted as homogeneous across all single-parent households, as pathways into single parenthood may differ in ways that are relevant to health and economic vulnerability.

The decomposition of expected expenditures clarifies that disparities in overall financial burden are driven primarily by differences in spending intensity rather than utilization probability. These results should be understood in light of how Table 4 was constructed: expected medical expenditure was calculated at the individual level as the product of the predicted probability from the first-part model and the predicted conditional mean expenditure from the second-part model, and the reported values are group averages of these individual-level predicted values. Single mothers experience a dual burden: elevated healthcare needs combined with higher conditional out-of-pocket spending. This pattern translates into the highest expected medical expenditure among all family types, underscoring a form of post-access vulnerability that universal coverage alone does not neutralize. This pattern is not unique to Korea; evidence from other East Asian universal insurance settings similarly shows that broad coverage does not necessarily eliminate unmet need or financial burden inequalities [27,28]. In this respect, our findings add to that literature by showing that the gap between formal coverage and effective financial protection is structured not only by socioeconomic vulnerability but also by the intersection of family structure and gender.

These results suggest that similar utilization rates do not imply equitable financial protection. Even in a system with near-universal insurance coverage, disparities remain evident through unmet need and differential exposure to medical spending. Family structure and gender are jointly associated with distinct patterns of disadvantage, highlighting the limitations of coverage expansion as a standalone policy solution.

From a policy perspective, these findings suggest that healthcare disparities under universal coverage do not arise uniformly across family types. For single mothers, policy priorities should include measures that reduce cost-sharing burdens, improve effective coverage for services with recurring care needs, and compensate for unequal access to supplementary private insurance among vulnerable family types. For single fathers, interventions aimed at lowering barriers to care initiation, including more flexible access to outpatient services and improved outreach, may be especially important. More broadly, addressing healthcare disparities among single-parent households may require coordination with wider social protection measures, particularly for families facing unstable employment and concentrated caregiving responsibilities. Addressing healthcare inequality in universal systems therefore requires moving beyond formal entitlement toward targeted measures that reduce cost-sharing burdens, improve effective coverage for services with recurring care needs, and compensate for unequal access to supplementary private insurance among vulnerable family types.

### 4.2. Limitations

Several limitations warrant careful consideration. First, although this study utilizes longitudinal panel data, the analysis remains observational in nature and does not establish causal relationships. The estimated associations between family structure and healthcare outcomes may be subject to selection bias. Individuals who become single parents may differ systematically from those in two-parent households in both observed and unobserved characteristics, including prior health status, socioeconomic trajectories, and psychosocial stress exposure. Such pre-existing differences may partially account for the disparities observed in healthcare utilization and expenditures.

Second, the possibility of reverse causality cannot be ruled out. Poor health may increase the likelihood of marital dissolution, separation, or reduced partnership stability, thereby influencing family structure. In this case, family structure would not be purely exogenous but partially shaped by underlying health conditions. Although we control for self-rated health and activity limitations, these measures may not fully capture long-term health selection processes.

Third, transitions into single parenthood—particularly through divorce or spousal bereavement—may involve acute health shocks and financial disruptions. The observed disparities may therefore reflect both structural vulnerability and the short- to medium-term consequences of marital transition events. Because the present analysis focuses primarily on between-group differences rather than event-based dynamics, it cannot disentangle these mechanisms.

Fourth, single-parent households are heterogeneous. The experiences of divorced, widowed, and never-married parents may differ substantially in terms of income stability, social support networks, and health trajectories. The Korea Health Panel does not allow for sufficiently granular differentiation of these subgroups across all waves, limiting our ability to examine within-category heterogeneity. Accordingly, the findings should be interpreted as average differences across broad household types rather than as uniform patterns applying equally to all single-parent households. Future research should distinguish pathways into single parenthood and incorporate event-history approaches to better understand how different family trajectories are associated with healthcare utilization and financial burden.

Fifth, although this study examines out-of-pocket expenditures as an indicator of financial burden, it does not assess catastrophic health expenditure using a threshold-based measure. As a result, the present analysis does not fully capture the extent to which healthcare spending may translate into severe economic hardship for vulnerable households.

Finally, family structure exhibits limited within-individual variation over time in this dataset, constraining the feasibility of fixed-effects estimation for the main models. Although fixed-effects estimates are reported as robustness checks, the reduced effective sample size limits statistical power. This issue is compounded by the relatively small number of observations for single-parent households, particularly single-father households, which may further reduce the precision of subgroup estimates. As a result, the findings should be interpreted as evidence of structural association rather than definitive causal effects.

## 5. Conclusions

This study finds that healthcare utilization and financial burden differ by family structure even within a universal health insurance system. By distinguishing between initiation of care and conditional spending, it clarifies stage-specific and gender-differentiated patterns of healthcare inequality.

The findings reveal pronounced gender-specific patterns. Single mothers do not differ significantly from two-parent households in initiating healthcare use, yet they experience higher unmet healthcare needs and incur greater medical expenditures once care is accessed. Higher conditional spending translates into the greatest expected financial burden among single mothers, suggesting a dual disadvantage rooted in health needs and economic vulnerability.

Single fathers, in contrast, face disadvantages primarily at the initiation stage. These contrasting patterns suggest gender-differentiated disparities in healthcare experiences among single-parent households.

Overall, similar utilization probabilities do not imply equal financial protection, and even under universal coverage, disparities remain evident across stages of healthcare utilization. Future research should incorporate threshold-based measures of catastrophic health expenditure to better evaluate financial protection and the risk of severe economic hardship across different family types.

## Figures and Tables

**Table 1:** Descriptive statistics by family structure.

Variable		Two-Parent Family (n = 53,309)		Single Mother (n = 3825)		Single Father (n = 752)		*p*-Value
		N	%	N	%	N	%	
Utilization of medical institutions	No	7348	13.79	458	11.98	116	15.43	<0.001
	Yes	45,946	86.21	3365	88.02	636	84.57	
Unmet healthcare needs	No	41,107	86.36	2710	78.10	543	81.04	<0.001
	Yes	6491	13.64	760	21.90	127	18.96	
Number of outpatient visits	Mean ± SD	14.69 ± 18.34		24.87 ± 27.98		27.24 ± 37.24		<0.001
Number of inpatient admissions	Mean ± SD	1.38 ± 1.10		1.85 ± 2.54		1.43 ± 0.97		<0.001
Medical expenditures (USD)	Mean ± SD	375.18 ± 811.51		546.06 ± 1130.11		447.50 ± 858.76		<0.001
Age (years)	Mean ± SD	47.93 ± 10.36		56.67 ± 12.08		57.50 ± 12.05		<0.001
	Range	19–97		25–94		27–98		
Educational attainment	Less than primary	3792	7.11	1320	34.51	156	20.74	<0.001
	Lower secondary education	4768	8.94	519	13.57	155	20.61	
	Upper secondary education	22,234	41.71	1397	36.52	300	39.89	
	Tertiary education	22,515	42.23	589	15.40	141	18.75	
Annual household income (USD)	Mean ± SD	38,316.90 ± 27,679.67		20,350.02 ± 15,049.64		23,337.57 ± 16,082.18		<0.001
Household size	2–3 household members	17,539	32.90	3601	94.14	695	92.54	<0.001
	4 household members	29,106	54.60	185	4.84	54	7.19	
	5 or more household members	6664	12.50	39	1.02	2	0.27	
Private health insurance enrollment	No	7930	14.88	1265	33.07	312	41.49	<0.001
	Yes	45,379	85.12	2560	66.93	440	58.51	
Self-rated health status	Very good	2342	5.14	108	3.21	29	4.48	<0.001
	Good	18,385	40.35	1020	30.32	189	29.21	
	Fair	20,434	44.85	1433	42.60	291	44.98	
	Poor	4100	9.00	709	21.08	113	17.47	
	Very poor	302	0.66	94	2.79	25	3.86	
Limitation in daily activities	No limitation	44,080	82.69	2881	75.32	566	75.27	<0.001
	Limited	9229	17.31	944	24.68	186	24.73	

Source: Korea Health Panel (KHP), 2010–2018 waves. Note: Monetary values were converted from Korean won (KRW) to U.S. dollars (USD) using a fixed exchange rate of 1 USD = 1450 KRW (as of 19 February 2026). Annual household income was originally reported in units of 10,000 KRW. Differences in age range across household types reflect the demographic composition of the analytic sample rather than differences in sample construction.

**Table 2:** Logit Regression Estimates of Healthcare Utilization and Unmet Healthcare Needs.

Variable		Healthcare Utilization		Unmet Healthcare Needs	
		OR	(95% CI)	OR	(95% CI)
Single father		0.50 **	(0.31–0.82)	1.19	(0.88–1.61)
Single mother		0.95	(0.72–1.25)	1.38 ***	(1.20–1.60)
ln(Age)		6.59 ***	(4.73–9.17)	0.56 ***	(0.45–0.68)
Educational attainment	Less than primary	REF	REF	REF	REF
	Lower secondary education	0.97	(0.67–1.41)	0.73 ***	(0.62–0.87)
	Upper secondary education	0.41 ***	(0.30–0.56)	0.70 ***	(0.60–0.81)
	Tertiary education	0.33 ***	(0.24–0.46)	0.67 ***	(0.57–0.79)
ln(Annual household income)		1.45 ***	(1.32–1.59)	0.72 ***	(0.67–0.77)
Household size	2–3 household members	REF	REF	REF	REF
	4 household members	0.68 ***	(0.60–0.78)	1.11 **	(1.02–1.21)
	5 or more household members	0.61 ***	(0.50–0.74)	1.23 ***	(1.08–1.40)
Private health insurance enrollment	No	REF	REF	REF	REF
	Yes	1.57 ***	(1.35–1.82)	1.00	(0.90–1.10)
Self-rated health status	Very good	REF	REF	REF	REF
	Good	1.24 ***	(1.07–1.44)	1.40 ***	(1.16–1.68)
	Fair	1.80 ***	(1.54–2.09)	2.25 ***	(1.87–2.70)
	Poor	3.11 ***	(2.48–3.90)	4.07 ***	(3.34–4.97)
	Very poor	5.14 ***	(2.50–10.57)	6.51 ***	(4.73–8.96)
Limitation in daily activities	No limitation	REF	REF	REF	REF
	Limited	1.00	(0.91–1.11)	0.98	(0.91–1.06)

Source: Korea Health Panel (KHP), 2010–2018 waves. Note: *** and ** denote statistical significance at the 1% and 5% levels, respectively.

**Table 3:** Regression Estimates of Utilization Intensity, Medical Expenditures, and Unmet Needs Among Healthcare Users.

Variable		Outpatient Visits	Inpatient Admissions	Medical Expenditures	Unmet Healthcare Needs
		*β*(SE)	*β*(SE)	*β*(SE)	OR(95% CI)
Single father		0.20 *** (0.05)	−0.08 (0.06)	0.03 (0.10)	1.24 (0.90–1.71)
Single mother		0.05 *** (0.02)	0.18 *** (0.07)	0.09 ** (0.04)	1.47 *** (1.27–1.71)
ln(Age)		0.95 *** (0.03)	0.16 *** (0.05)	0.83 *** (0.05)	0.52 *** (0.42–0.64)
Educational attainment	Less than primary	REF	REF	REF	REF
	Lower secondary education	−0.11 *** (0.02)	0.00 (0.04)	−0.04 (0.03)	0.75 *** (0.63–0.89)
	Upper secondary education	−0.24 *** (0.02)	0.05 (0.05)	−0.08 ** (0.03)	0.71 *** (0.61–0.83)
	Tertiary education	−0.36 *** (0.02)	−0.03 (0.05)	−0.15 *** (0.04)	0.70 *** (0.60–0.83)
ln(Annual household income)		0.01 (0.01)	0.01 (0.02)	0.25 *** (0.02)	0.73 *** (0.68–0.78)
Household size	2–3 household members	REF	REF	REF	REF
	4 household members	−0.11 *** (0.01)	0.01 (0.02)	−0.22 *** (0.02)	1.12 ** (1.03–1.23)
	5 or more household members	−0.08 *** (0.02)	0.12 ** (0.05)	−0.23 *** (0.03)	1.23 *** (1.07–1.40)
Private health insurance enrollment	No	REF	REF	REF	REF
	Yes	0.01 (0.01)	0.01 (0.03)	0.16 *** (0.03)	1.00 (0.90–1.12)
Self-rated health status	Very good	REF	REF	REF	REF
	Good	0.09 *** (0.03)	0.02 (0.03)	0.09 * (0.05)	1.38 *** (1.12–1.69)
	Fair	0.32 *** (0.03)	0.06 ** (0.03)	0.29 *** (0.05)	2.21 *** (1.80–2.71)
	Poor	0.76 *** (0.03)	0.30 *** (0.04)	0.81 *** (0.05)	4.07 *** (3.28–5.06)
	Very poor	0.97 *** (0.06)	0.66 *** (0.09)	1.12 *** (0.09)	6.35 *** (4.55–8.88)
Limitation in daily activities	No limitation	REF	REF	REF	REF
	Limited	0.09 *** (0.01)	0.11 *** (0.03)	0.11 *** (0.03)	1.00 (0.92–1.09)

Source: Korea Health Panel (KHP), 2010–2018 waves. Note: ***, **, and * denote statistical significance at the 1%, 5%, and 10% levels, respectively. Outpatient and inpatient visit counts are estimated using negative binomial models; medical expenditures are estimated using a gamma generalized linear model (GLM) with a log link; unmet healthcare needs are reported as odds ratios (ORs) with 95% confidence intervals among healthcare users (MedicalUse = 1).

**Table 4:** Decomposition of expected out-of-pocket expenditures by family type.

Category	Probability of Medical Utilization ($\hat{P}$, %)	Predicted Conditional Mean Expenditure ($\hat{\mu }$, USD)	Expected Medical Expenditure per Person ( $\hat{E}$, USD)	
Two-parent family	86.66	433.79	384.22	
Single father	86.33	574.13	507.91	
Single mother	90.58	607.85	561.90	

Source: Korea Health Panel (KHP), 2010–2018 waves; authors’ calculations. Note: Monetary values originally reported in Korean won (KRW) were converted to U.S. dollars (USD) using a fixed exchange rate of 1 USD = 1450 KRW (as of 19 February 2026). All entries represent averages of individual-level predicted values over the full analytic sample.

## Data Availability

The data that support the findings of this study are available from the Korea Health Panel (KHP). Restrictions apply to the availability of these data, which were used under license for the current study and are not publicly available. Data are available from the Korea Health Panel upon reasonable request and with permission of the data provider.

## Abbreviations

The following abbreviations are used in this manuscript:

KHP	Korea Health Panel
GLM	Generalized Linear Model
OECD	Organisation for Economic Co-operation and Development
USD	United States Dollar

## Appendix A

**Table A1:** Fixed-effects logit estimates.

Variable		Healthcare Utilization		Unmet Healthcare Needs	
		OR	(95% CI)	OR	(95% CI)
Single father		0.60	(0.25–1.47)	1.52	(0.75–3.07)
Single mother		0.82	(0.46–1.48)	0.98	(0.66–1.43)
ln(Age)		1.05 ***	(1.04–1.06)	0.96 ***	(0.96–0.97)
Educational attainment	Less than primary	REF	REF	REF	REF
	Lower secondary education	1.91	(0.20–17.93)	0.27 *	(0.07–1.08)
	Upper secondary education	0.86	(0.13–5.98)	0.72	(0.22–2.39)
	Tertiary education	1.30	(0.18–9.66)	0.54	(0.15–1.96)
ln(Annual household income)		1.09	(0.96–1.24)	0.98	(0.88–1.10)
Household size	2–3 household members	REF	REF	REF	REF
	4 household members	0.59 ***	(0.48–0.71)	1.04	(0.88–1.22)
	5 or more household members	0.55 ***	(0.37–0.82)	0.97	(0.69–1.38)
Private health insurance enrollment	No	REF	REF	REF	REF
	Yes	1.13	(0.90–1.43)	1.12	(0.89–1.40)
Self-rated health status	Very good	REF	REF	REF	REF
	Good	1.08	(0.93–1.26)	1.29 **	(1.06–1.59)
	Fair	1.32 ***	(1.12–1.55)	1.93 ***	(1.58–2.37)
	Poor	1.49 ***	(1.17–1.91)	2.93 ***	(2.34–3.68)
	Very poor	2.34 *	(0.98–5.62)	4.41 ***	(3.02–6.47)
Limitation in daily activities	No limitation	REF	REF	REF	REF
	Limited	0.98	(0.88–1.08)	0.94	(0.86–1.02)

Note: Odds ratios (OR) are reported. Fixed-effects logit models are estimated using only individuals with within-person variation in the outcome; panels with all-0 or all-1 outcomes are excluded. ln(Age) is scaled so that the reported OR corresponds to a 0.01 increase in ln(Age). Significance levels: *** *p* < 0.01, ** *p* < 0.05, * *p* < 0.10. Healthcare utilization FE sample: 2973 individuals (7151 excluded). Unmet healthcare needs FE sample: 4000 individuals (6124 excluded).
